# On the Mode of Administering Opium in Delirium Tremens

**Published:** 1872-01

**Authors:** 


On the Mode of Administering Opium in Delirium Tremens. It is stated (*Echo Medical et Pharmaceutique Belge*, 1871,) that spontaneous recovery is the natural mode of termination of delirium tremens in drunkards. This may be true, but there is a number of cases in which expectation would be dangerous, especially those in which the delirium is complicated by a fracture or some severe injury. Moreover, why not, when one is able, put an end to scenes of violence which are similar to those which render so dangerous the subjects of acute mania? Dupuytren, in order to arrest the nervous delirium of operation subjects, or of injured patients suspected of drinking habits, employed small enemata, containing six, eight or ten drops of laudanum. If the first injection be rejected, a second, and, if necessary, a third is used. If the patient be put to sleep, his condition promises well, and the treatment is discontinued for twenty-four hours. If the calm be an incomplete one, a fresh enema is given, and repeated every six hours. The reasons given by Dupuytren for preferring the rectum to the stomach, for the administration of opium in these cases, are the following: The rectum absorbs, and does not digest the medicinal agent, which, therefore, passes more directly and more surely to its destination; six drops of laudanum, administered in an injection, take more effect than a gramme of the same preparation given by the mouth. Besides, it is often a difficult matter to get the subject of delirium tremens to swallow.

